# The risk factors for insomnia and sleep-disordered breathing in military communities: A meta-analysis

**DOI:** 10.1371/journal.pone.0250779

**Published:** 2021-05-06

**Authors:** Yujia Huang, Jingzhou Xu, Siqi Zheng, Shuyu Xu, Yajing Wang, Jing Du, Lei Xiao, Ruike Zhang, Hao Wang, Yunxiang Tang, Tong Su

**Affiliations:** 1 Department of Medical Psychology, Faculty of Psychology, Naval Medical University (Second Military Medical University), Shanghai, China; 2 Department of Psychology, TongJi University, Shanghai, China; Harper University Hospital, UNITED STATES

## Abstract

**Background:**

Many reviews and meta-analyses had been conducted to investigate risk factors for sleep disorders in the general population. However, no similar research has been performed in the military population though insomnia and sleep-disordered breathing are quite prevalent in that population.

**Objectives:**

To investigate risk factors for insomnia and sleep-disordered breathing in military personnel.

**Methods:**

A systematic literature search was performed from inception to March 2021 and 6496 records were produced. Two authors independently screened records for eligibility. Results were presented as odds ratios, and a random-effect model was used to pool results. Data analysis was performed respectively according to military personnel type (i.e., veteran, active-duty personnel). Risk factors were sorted into three categories: sociodemographic, army-specific, and comorbidity. This meta-analysis was registered in PROSPERO before data analysis (registration No: CRD42020221696).

**Results:**

Twenty-seven articles were finally included in the quantitative analysis. For sleep-disordered breathing in active-duty personnel, four sociodemographic (i.e., overweight/obesity, higher body mass index, male gender, >35 years old) and one comorbidity (i.e., depression) risk factors were identified. For insomnia in active-duty personnel, four sociodemographic (i.e., aging, alcohol dependence, white race, and female gender), two army-specific (i.e., deployment experience, combat experience), and four comorbidity (i.e., depression, post-traumatic stress disorder, traumatic brain injury, and anxiety) risk factors were identified. For insomnia in veterans, one army-specific (i.e., combat experience) and one comorbidity (i.e., post-traumatic stress disorder) risk factor was identified.

**Conclusions:**

Several risk factors were identified for insomnia and sleep-disordered breathing in the current meta-analysis. Risk factors for veterans and active-duty personnel were partially different. Research on sleep breathing disorders remains limited, and more convincing evidence would be obtained with more relevant studies in the future.

## Introduction

Healthy sleep requires good sleep quality, adequate sleep duration, regular circadian rhythm, and absence of sleep disorders. Military personnel have more difficulties fulfilling these requirements, because they need to keep up with their daily training and carry out deployment or combat tasks. Military tasks are always of high intensity and require the personnel to operate under extreme and harsh conditions, including cold stress [1], heat stress [2], and high altitude [3]. When deployed, the disturbed time schedule and latent danger place extreme pressure on soldiers, damaging their daily sleep patterns. A newly formed 5 h on/10 h off time schedule on navy ships results in poor sleep hygiene and abundant sleep debt [4]. Consensus has been reached that adults should sleep at least seven hours per night on a regular basis to maintain health whereas sleep need increases to nine hours in adolescence and early adulthood [5, 6]. This age range coincides with active-duty military personnel [7]. However, it is quite common for military personnel to sleep less than six hours per night [8]. A study on militarily relevant tasks after long-term sleep loss finds that mental-effort-requiring tasks were more influenced than those requiring only physical energy [9].

The prevalence of sleep disorders are surprisingly high in military personnel [10]. Insomnia and sleep-disordered breathing (SDB) are quite prevalent in the military [11–14]. Insomnia is defined as difficulties initiating or maintaining sleep or waking up too early and inability to return to sleep, accompanied by fatigue during wakefulness [15]. The common diagnosis tool for insomnia is the International Classification of Diseases and International Classification of Sleep Disorders. The Insomnia Severity Index (ISI) [16], a self-reported questionnaire, is also widely used for identifying insomnia with higher scores indicating more severe insomnia symptoms. Insomnia could cause serious harm to the military as it can affect cognitive functions such as working memory [17], executive function [17], and declarative memory [18] and increase the risk for motor vehicle accidents among military personnel [19]. SDB, defined as breathing problems during sleep, includes obstructive sleep apnea (OSA), central sleep apnea (CSA), sleep-related hypoventilation disorders, and sleep-related hypoxemia disorder [20–22]. Growing evidence indicates that apnea and subsequent compensatory hyperpnea could have adverse cardiovascular consequences [23], and OSA has a positive correlation with death [24]. SA could weaken working memory, executive functions, and many other aspects of cognition ability [25].

Potential sociodemographic risk factors for developing SDB and insomnia are of vital importance because officers could know in advance which population is at higher risk for developing certain sleep disorders and implement early intervention for high-risk groups. Military personnel are more likely to experience traumatic events during deployment and develop comorbidities such as post-traumatic stress disorder (PTSD) [26] and depression [27] as a result. Poorer sleep quality [28] and higher prevalence of OSA [29] were found in patients with PTSD. It had been widely agreed that depression can lead to sleep impairment. Traumatic brain injury (TBI) could result in poorer sleep quality [30] and higher prevalence of insomnia [31]. Therefore, this study planned to examine three major categories of risk factors: sociodemographic, army-specific, and comorbidity.

Young and Punjabi [32, 33] reviewed risk factors for OSA in a nonmilitary sample. Risk factors for SDB in the general population are obesity [32], snoring [34, 35], aging [32, 36, 37], and cardiovascular factors such as hypertension [38, 39], smoking [32, 33], and alcohol dependence [33]. Ohayon [31] reviewed the epidemiology of insomnia in the general sample. Notwithstanding, there was no systematic review focusing on risk factors for sleep disorders in the military to our best knowledge.

We aimed to investigate risk factors (i.e., sociodemographic, army-specific, and comorbidity) for insomnia and SDB in the military.

## Materials and methods

The literature search process was performed according to the Preferred Reporting Items for Systemic Reviews and Meta-Analysis (PRISMA) checklist [40] (S2 File) and was registered with PROSPERO (S1 File) before the data analysis (registration No: CRD42020221696).

### Database searching

PubMed, Embase, PsycINFO, and Web of Science were searched from inception to April 2020, limited to papers published in the English language. Search terms were as follows: sleep OR sleep problem OR sleep disorder OR sleep disturbance AND veteran* OR soldier* OR army OR navy OR marine OR troop OR air force OR armed OR peacekeeper* OR defense AND risk OR predictor* OR prediction OR predisposition. The full search strategy is presented in S3 File. In all, 5105 records were identified through searching while another nine were included by hand-searching the references of meta-analyses and reviews in this area. The search was further updated in March 2021 to ensure the completeness of our search scope.

### Eligibility criteria

Four independent authors (Huang, Xu, Meng, Li) reviewed all titles and abstracts to determine eligibility. Full texts were screened when eligibility could not be determined by the titles or abstract alone, and any discrepancies were resolved by consensus to identify articles that met the following inclusion criteria: (a) investigating risk factors for insomnia and SDB in military populations; (b) including a sample of military personnel, veterans, or both; (c) containing effect sizes such as risk ratio (RR), odds ratio (OR), hazard ratio (HR) and corresponding 95% confidence interval (CI), or standard error (SE) or data able to calculate the above parameters; (d) study design: cohort, case-control, or cross-sectional.

The exclusion criteria were as follows: (a) including both active-duty personnel and veterans while data of each group cannot be extracted separately; (b) having no controls or comparative group who are not exposed to studied risk factor; (c) not containing data essential to calculate needed effect sizes and cannot be obtained from authors; (d) using continuous data to describe sleep disorder severity; (e) not published in English.

### Data extraction

A Standardized Excel form was used by two independent authors to reduce probable errors. The data extraction form involved (a) general information: study design, country, subject type, male proportion, average age, and standard deviation (SD), sleep disorder type, diagnostic method, etc.; (b) sociodemographic factors: body mass index (BMI), marital status, race, gender, age, education level, alcohol dependence, smoking, income, etc.; (c) army-specific factors: rank, combat experience and time, deployment experience and time, military experience, etc.; and (d) comorbidities: PTSD, TBI, depression, anxiety, etc. The above factors and corresponding events and totals for cases and controls or calculated effect sizes such as ORs, RRs, or HRs were extracted. Crude effect sizes were extracted to reduce the bias brought by different confounders of different studies. Adjusted effect sizes were included when they were the only results.

### Outcome measurement

The primary outcomes of this meta-analysis were risk factors for insomnia and SDB in military communities. The association between sleep disorders and associated factors was assessed using OR. Therefore, the merged results were all presented as OR and 95%CI.

### Study quality assessment

Two authors (Huang, Xu) assessed the quality of the included studies separately. The Newcastle-Ottawa Scale, one of the most commonly used tools for assessing methodological quality of non-randomized studies, was utilized to assess the quality of included cohort and case control studies. Each article was evaluated from three aspects (i.e., selection, comparability, exposure/outcome) including eight detailed questions. The Agency for Healthcare Research and Quality was utilized to assess cross-sectional studies. An answer of “NO or “UNCLEAR” is scored “0”, and “YES” is scored “1”.

### Statistical analysis

We examined risk factors for insomnia and SDB in military personnel based on OR or original data, which include number of participants exposed and not exposed to risk factors and number of participants with sleep disorders in the two groups. Considering variation in the diagnostic tools, participant characteristics, and study type of the included articles, a random-effect model was used to estimate the pooled ORs with 95% CIs. I^2^ statistic was used to measure heterogeneity with I^2^ values below 25%, 50%, and 75% representing low, moderate, and high level of heterogeneity, respectively. Further subgroup and sensitivity analyses to control heterogeneity level were not feasible as most of the outcomes contained limited number of studies due to detailed sorting based on their characteristics.

Data were analyzed using Revman 5.4 and Stata 16. For studies only providing continuous data for probable factors (e.g., specific BMI instead of obese or not) and those still meaningful after being transferred into ORs, standard mean difference (SMD) was transferred into OR based on the assumption that continuous variables within two intervention groups have equal SD logistic distributions [41, 42], using the following equation:

$$\ln\mathrm{OR}=\frac{\pi }{\sqrt{3}}\mathrm{SMD}$$

The corresponding SE can be calculated from 95%CI by the following equation:

SE=(upperlimit−lowerlimit)/3.92

Outliers were identified by visually checking the forest plots, and those that do not overlap with the 95%CI of pooled effect size were removed from the quantitative analysis.

As for publication bias assessment, for risk factors containing more than 10 articles, funnel plot was used to assess publication bias. For those containing less than 10 but more than 2 articles, Egger’s test was utilized in factors using lnOR and corresponding SE to merge results, and Harbord’s test was utilized in factors using original data (i.e., number of participants exposed or not exposed to risk factor and corresponding number of participants developing targeted sleep disorder) to merge results.

## Results

### Study selection

**Fig 1:**
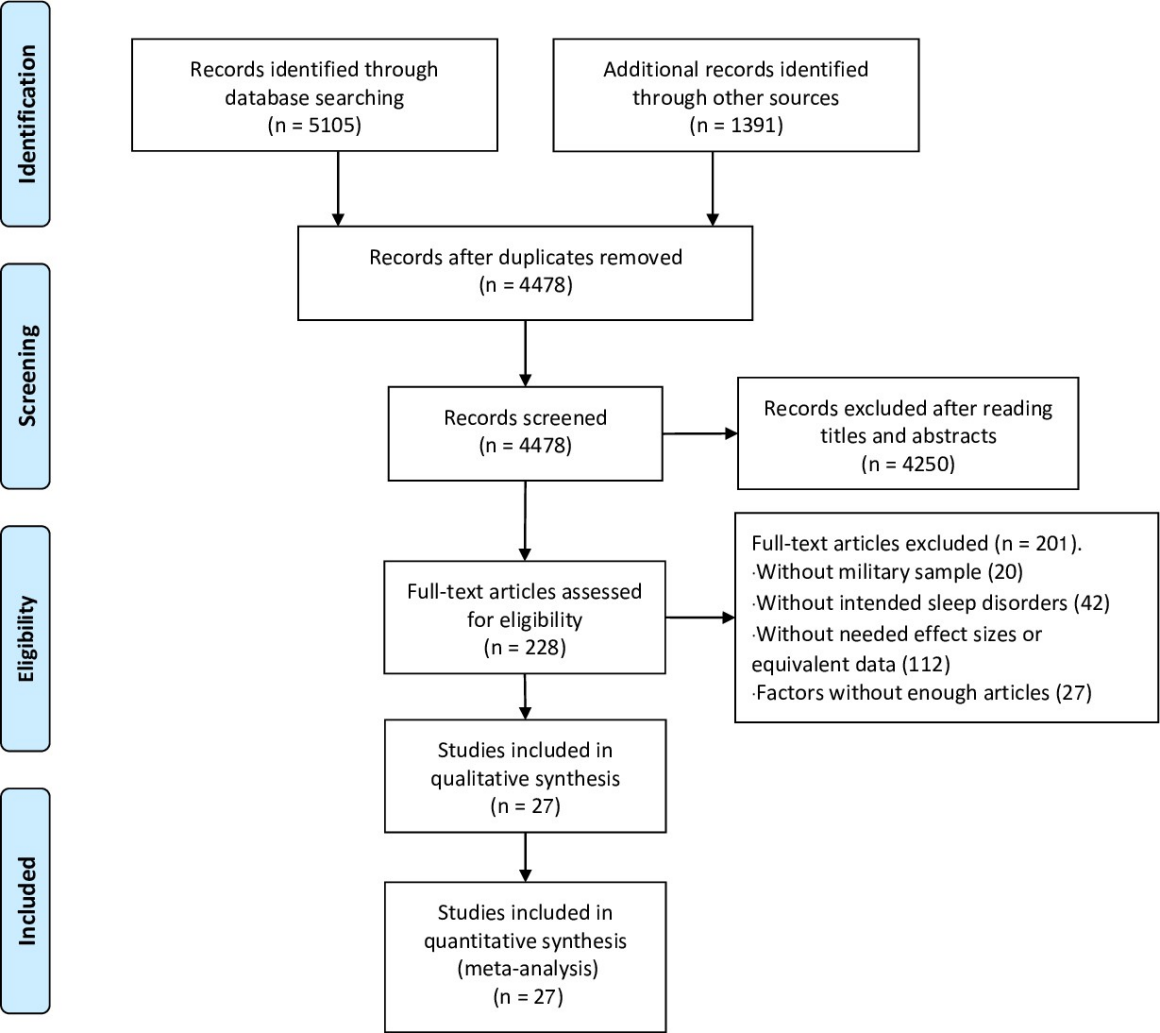
PRISMA flow-chart.

PubMed, Embase, PsycINFO, and Web of Science were searched from inception to April 2020 with 5105 records obtained. Nine records were included by hand-searching the references of meta-analyses and reviews in this area; 3714 records remained after the removal of duplications. Two authors independently screened the titles and abstracts of the remaining studies and excluded 3530 studies that did not meet eligibility requirements (i.e., no military participants, irrelevant of sleep disorders, no needed effect sizes). One hundred eighty-four full texts were read independently by two trained assistants; 139 records were excluded for not meeting the inclusion criteria. Twenty-five records were further excluded since their studying factors did not contain a sufficient volume of literature to perform meta-analysis. Another three records were excluded for mixed data for veterans and active-duty personnel with 17 articles included for quantitative analysis in the first stage of search. We updated our search in March 2021, included another 1382 records, and included 10 more articles for quantitative analysis after screening. As a result, a total of 27 articles were included in this meta-analysis. See the process in Fig 1.

### Study characteristics

Detailed information of the 27 selected articles is presented in Table 1. The detailed information of quality assessment is provided in S4 File. No publication bias exist except for one factor (i.e., white race for insomnia risk in veterans) (S5 File). Thirteen studies explored SDB’s risk factors, and 19 explored risk factors for insomnia. SDB patients were diagnosed using the Berlin Questionnaire (BQ) in two studies [34, 43], the 9^th^ and 10^th^ edition of ICD in three studies [44–46], the 2^nd^ or 3^rd^ edition of ICSD in two studies [47, 48], the Apnea Hypopnea Index (AHI) in four studies [47–50], previously documented information in two studies [51, 52], self-reported SDB in one study [53], and no diagnose tool in one study [54]. As for the definition of insomnia, two studies used the 9^th^ and 10^th^ edition of ICD [44, 55], three used the 2^nd^ and 3^rd^ edition of ICSD [47, 48, 56], seven used the ISI [48, 57–62], one used the Brief Insomnia Questionnaire (BIQ) [63], four used a self-edited questionnaire [63–66], two used self-reported data [53, 67], and one used documented information [52].

**Table 1:** Study characteristics.

Included study	Study design	Participant type	Male/Female	Age	Disorder type	Diagnose tool
				M (SD)		
Baird et al. 2018 [51]	cross-sectional	Veterans	214/0	69 (4)	SDB^a^	Documented diagnosis
Cairns et al. 2017 [49]	cross-sectional	Veterans	1370/130	52.8 (13.5)	SDB^a^	AHI^d^
Crump et al. 2019 [45]	cohort	Active-duty personnel	1547478/0	18	SDB^a^	ICD^e^-10
Langton et al. 2016 [46]	cohort	Active-duty personnel	467885/	/	SDB^a^	ICD^e^-9
Lee et al. 2013 [34]	cross-sectional	Active-duty personnel	665/0	21.76 (1.19)	SDB^a^	BQ^c^
Mysliwiec et al. 2015 [54]	cross-sectional	Active-duty personnel	106/3	34.3 (8.23)	SDB^a^	/
Pilakasiri et al. 2018 [43]	cross-sectional	Active-duty personnel	1036/71	26.9 (8)	SDB^a^	BQ^c^
Iqbal et al. 2016 [50]	cross-sectional	Veterans	214/0	63 (6)	SDB^a^	AHI^d^
Mysliwiec et al. 2013 [68]	cross-sectional	Active-duty personnel	676/49	35.5 (8.6)	SDB^a^, Insomnia	AHI^d^, ICSD^g^-2
Martindale et al. 2020 [52]	cross-sectional	Veterans	256/37	41.63 (10.14)	SDB^a^, Insomnia	Documented diagnosis
Caldwell et al. 2019 [44]	cohort	Active-duty personnel	/	/	SDB^a^, Insomnia	ICD^e^-9
Foster et al. 2017 [48]	cross-sectional	Active-duty personnel	108/101	34.3 (8.52)	SDB^a^, Insomnia	ICSD^g^-3, ISI^h^, AHI^d^
Kanefsky et al. 2019 [69]	cross-sectional	Active-duty personnel	113/5	32.82 (7.73)	SDB^a^, Insomnia	Self-reported
Hermes et al. 2014 [55]	cross-sectional	Veterans	5037101/492099	61.2 (16.3)	Insomnia	ICD^e^-9
Klingaman et al. 2017 [63]	cross-sectional	Active-duty personnel	18790/2514	27 (7.4)	Insomnia	SEQ^f^
Lopez et al. 2013 [64]	cross-sectional	Veterans	144/22	56 (15)	Insomnia	SEQ^f^
Martin et al. 2017 [56]	cross-sectional	Veterans	0/660	50.9 (17.7)	Insomnia	ICSD^g^-2
Taylor et al. 2016 [57]	cross-sectional	Active-duty personnel	3719/359	27.43 (6.14)	Insomnia	ISI^h^
Pettersson et al. 2016 [65]	cross-sectional	Veterans	966/115	36.1 (9.9)	Insomnia	SEQ^f^
Adrian et al. 2018 [58]	cross-sectional	Active-duty personnel	2640/239	/	Insomnia	ISI^h^
Colvonen et al. 2020 [59]	cross-sectional	Veterans	4597/955	34.81 (9.07)	Insomnia	ISI^h^
Mosti et al. 2019 [70]	cross-sectional	Active-duty personnel	580023/90524	29.5 (7.5)	Insomnia	BIQ^i^
King et al. 2017 [60]	cohort	Veterans	247/21	31 (8)	Insomnia	ISI^h^
Scoglio et al. 2017 [61]	cross-sectional	Veterans	118/46	35.15 (9.19)	Insomnia	ISI^h^
Sandman et al. 2013 [67]	cross-sectional	Veterans	23005/23669	/	Insomnia	Self-reported
Hu et al. 2020 [66]	cross-sectional	Veterans	1025/49	/	Insomnia	SEQ^f^
Gaffey et al. 2020 [62]	cross-sectional	Veterans	534/575	43.8 (10.9)	Insomnia	ISI^h^

Note

a: Sleep-disordered breathing

b: Neurobehavioral Symptom Inventory

c: Berlin Questionnaire

d: Apnea Hypopnea Index

e: International Classification of Diseases

f: Self-edited questionnaire

g: International Classification of Sleep Disorders

h: Insomnia Severity Index

i: Brief Insomnia Questionnaire

/: no corresponding data.

The total sample consisted of 21,923,699 in the military population and 28,223 in the general population. The percentage of male participants was 91.73% in the military population and 45.93% in the general population. The ages ranged from less than 20 years old to more than 75 years old. Of the 27 studies, 13 included a total of 16,335,530 active-duty military personnel while the remaining 14 included a total of 5,588,169 veterans. Twenty-one studies were in the U.S., and the remaining six were in Australia (one), Sweden (two), Korea (one), Thailand (one), and Finland (one). All studies were written in English. Publish dates ranged from 2013 to 2020. Extracted factors had to contain a minimum of two articles to be entered into the analysis. Final presented outcomes were divided into three groups: sociodemographic, army-specific, and comorbidities. Information was mostly collected through network or questionnaire. Risk or preventive factors for SDB and insomnia in active-duty personnel or veterans were discussed, respectively.

### Risk factors for SDB in active-duty personnel

#### Sociodemographic factors

**Fig 2:**
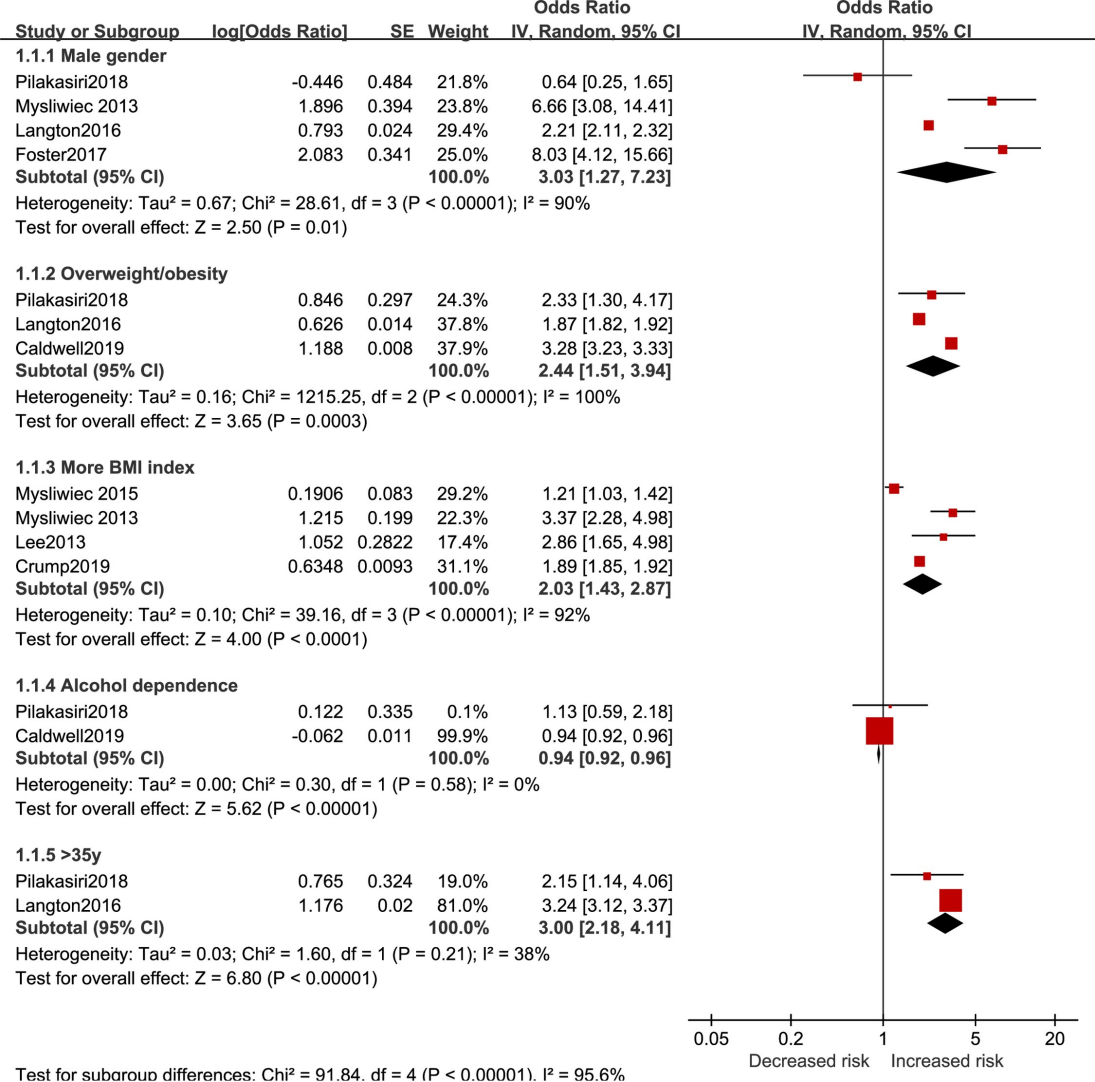
Sociodemographic factors for SDB in active-duty personnel.

The results of sociodemographic effect on SDB risk in active-duty personnel are reported in Fig 2. SDB risk was significantly higher in the overweight and obese population (OR = 2.44, 95%CI 1.51 to 3.94), and higher BMI was associated with higher risk (OR = 2.03, 95%CI 1.43 to 2.87). Being male (OR = 3.03 95%CI 1.27 to 7.23) and older than 35 years old (OR = 3.00 95%CI 2.18 to 4.11) could increase SDB risk as well. Results showed that alcohol dependence led to a slight but significant decrease of risk (OR = 0.94, 95%CI 0.92 to 0.96).

#### Comorbidity factors

**Fig 3:**
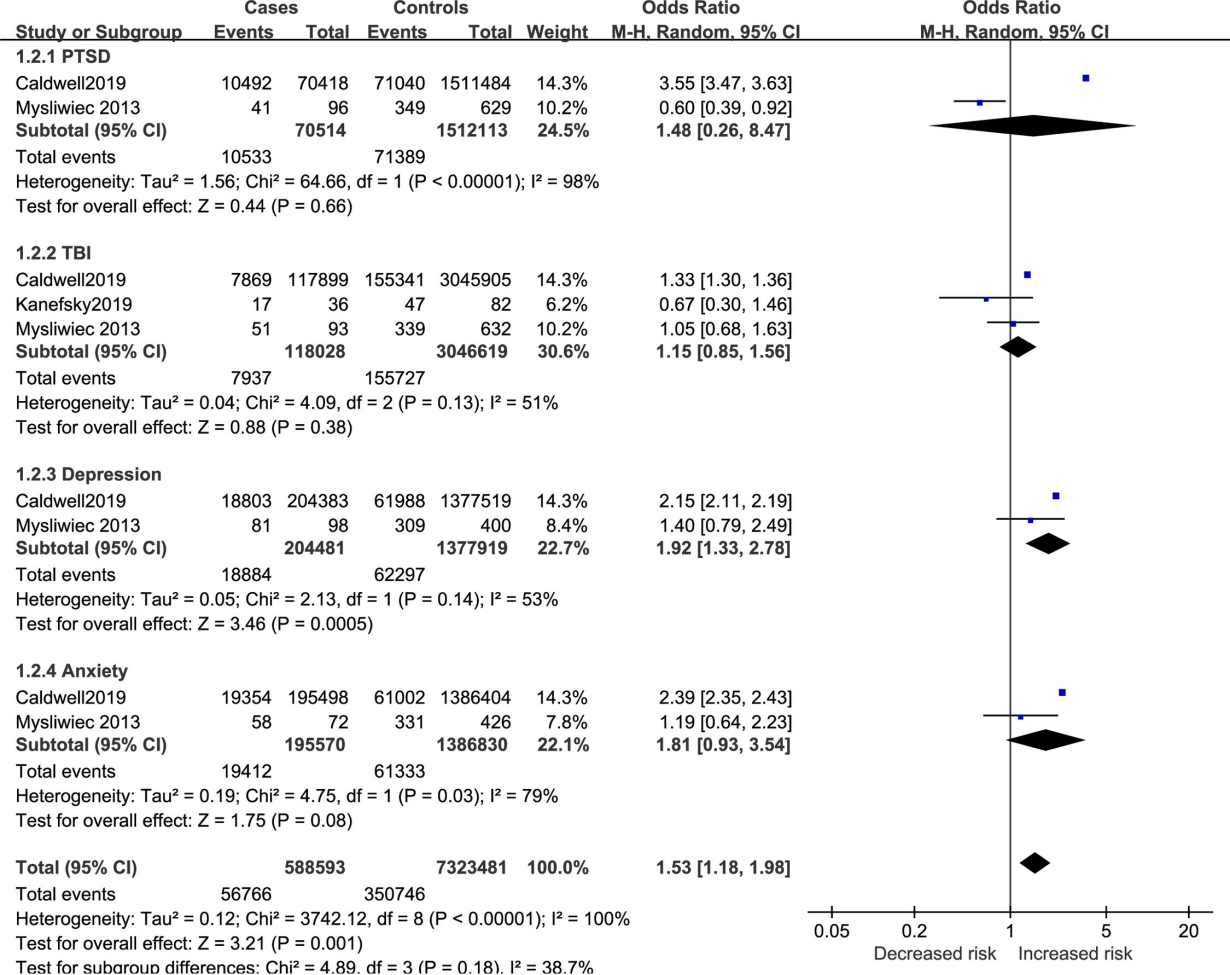
Comorbidity factors for SDB in active-duty personnel.

Results of comorbidity effect on SDB risk in active-duty personnel are reported in Fig 3. Depression was found to increase SDB risk (OR = 1.92, 95%CI 1.33 to 2.78) while PTSD (OR = 1.48, 95%CI 0.26 to 8.47), TBI (OR = 1.15, 95%CI 0.85 to 1.56), and anxiety (OR = 1.81, 95%CI 0.93 to 3.54) had no influence on SDB risk. The pooled result of the four comorbidities showed significant increase for SDB risk in active-duty personnel (OR = 1.53, 95%CI 1.18 to 1.98).

### Risk factors for SDB in veterans

#### Comorbidity factor

**Fig 4:**
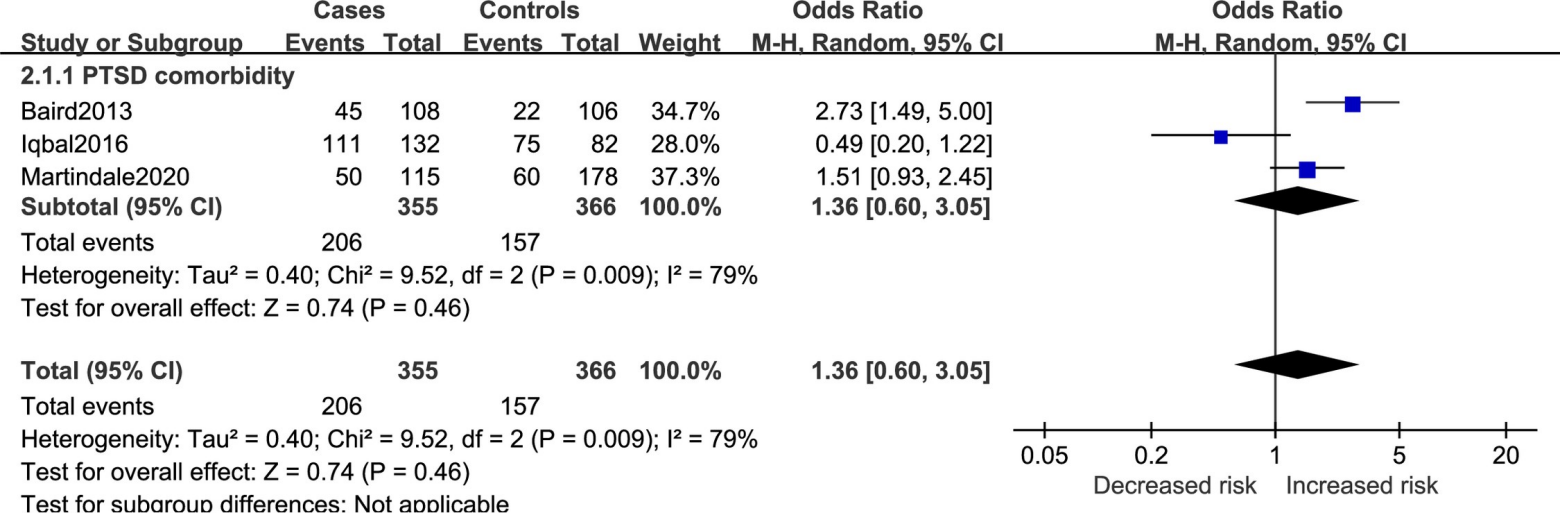
Comorbidity factor for SDB in veterans.

Results regarding the comorbidity factor for SDB in veterans is reported in Fig 4. PTSD had no significant influence on SDB risk (OR = 1.36; 95%CI 0.60 to 3.05).

### Risk factors for insomnia in active-duty personnel

#### Sociodemographic factors

**Fig 5:**
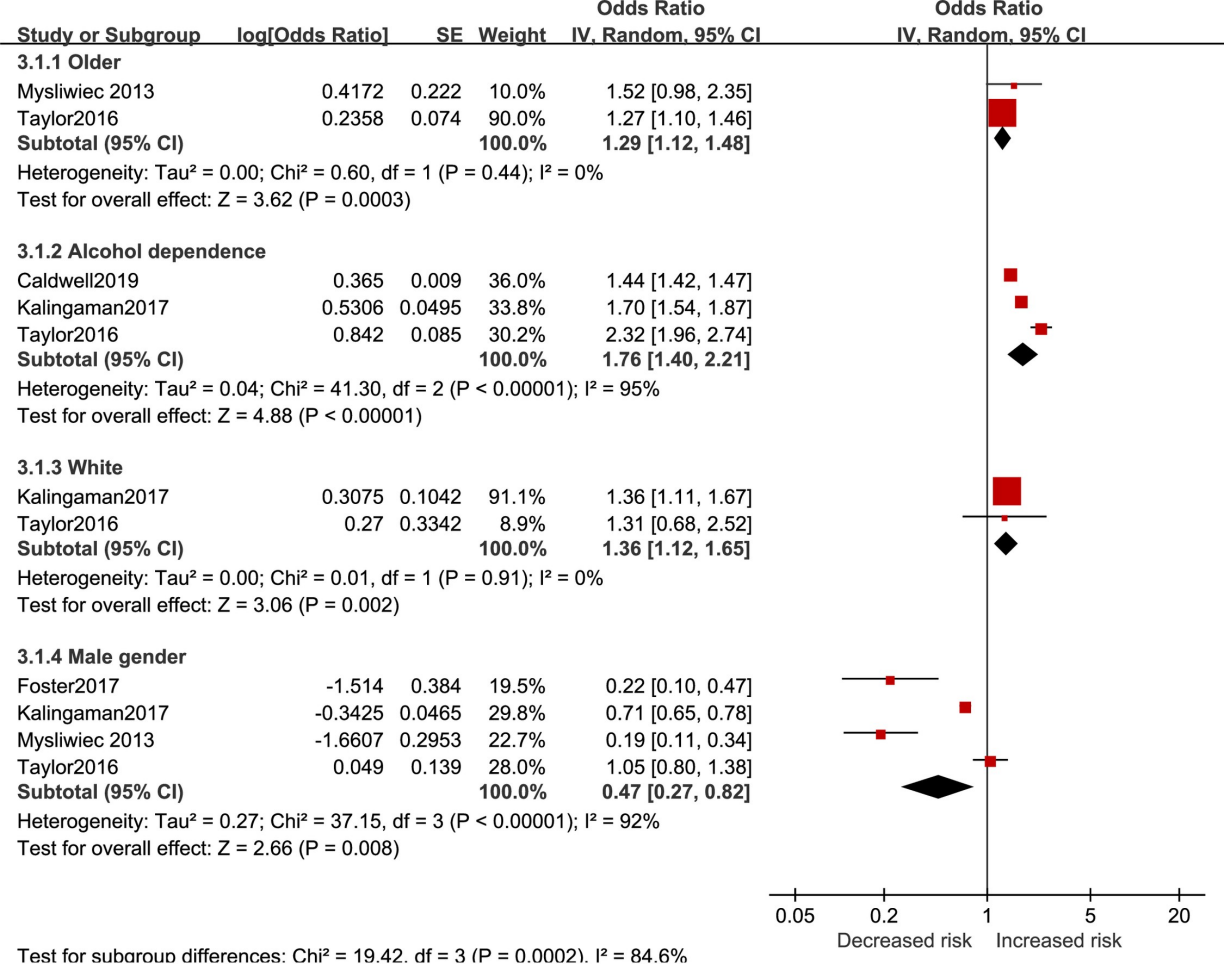
Sociodemographic factors for insomnia in active-duty personnel.

Results regarding the sociodemographic effect on insomnia risk in active-duty personnel are reported in Fig 5. Aging (OR = 1.29 95%CI 1.12 to 1.48), having alcohol dependence (OR = 1.76 95%CI 1.40 to 2.21), and being white (OR = 1.36 95%CI 1.12 to 1.65) showed higher risk for insomnia. Males participants were at lower risk for developing insomnia (OR = 0.47 95%CI 0.27 to 0.82).

##### Army-specific factors

**Fig 6:**
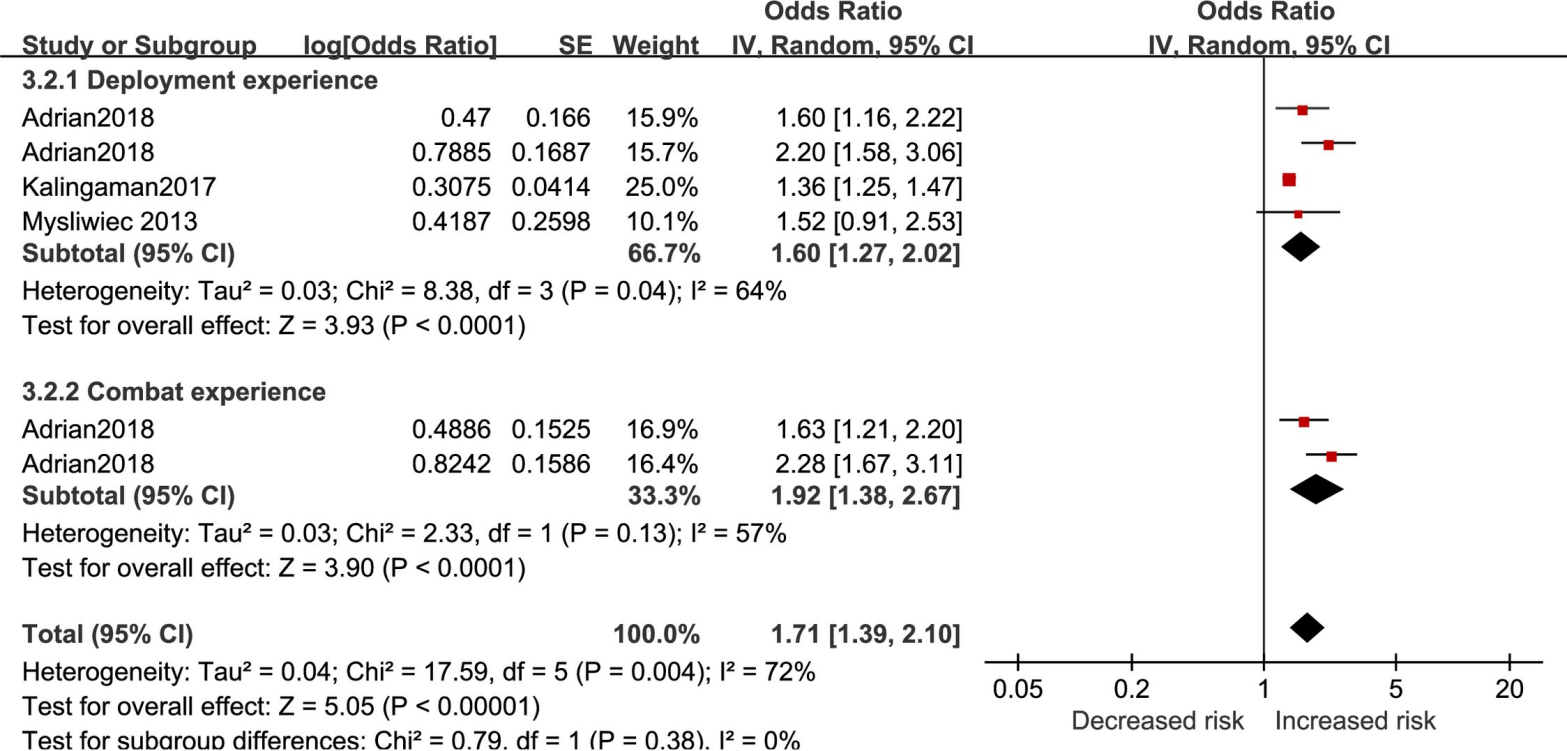
Army-specific factors for insomnia in active-duty personnel.

Results of army-specific effect on insomnia risk are reported in Fig 6. Deployment experience (OR = 1.60, 95%CI 1.27 to 2.02) and combat exposure (OR = 1.92, 95%CI 1.38 to 2.67) led to higher insomnia risk. The total effect of army-specific factors showed a significant increase of insomnia risk (OR = 1.71, 95%CI 1.39 to 2.10).

#### Comorbidity factors

**Fig 7:**
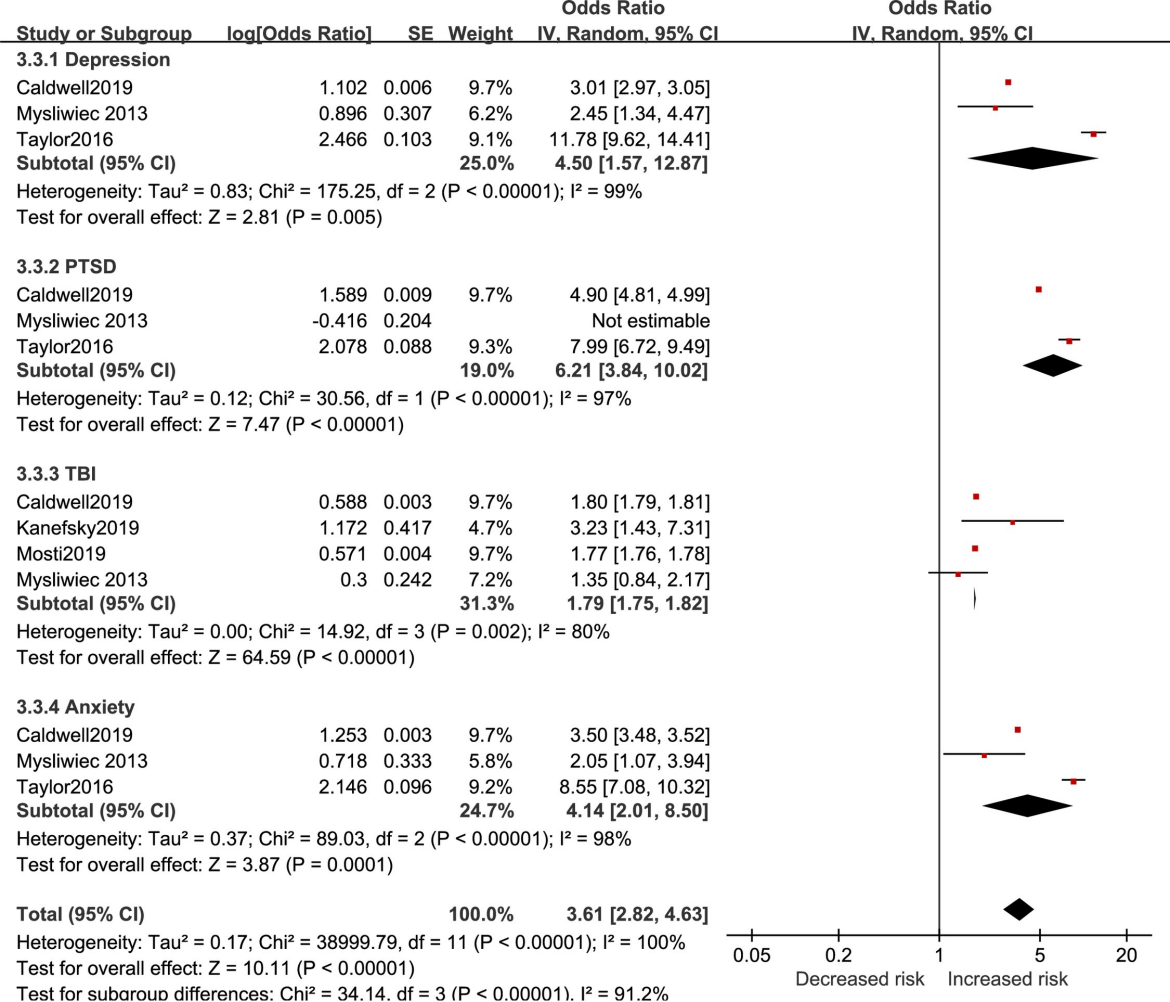
Comorbidity factors for insomnia in active-duty personnel.

Results of comorbidity effect for insomnia risk are reported in Fig 7. One included article in the PTSD category was identified as an outlier and excluded during data analysis [13]. Depression (OR = 4.50, 95%CI 1.57 to 12.87), PTSD (OR = 6.21, 95% 3.84 to 10.02), TBI (OR = 1.79, 95%CI 1.75 to 1.82), and anxiety (OR = 4.14, 95%CI 2.01 to 8.50) could increase insomnia risk. Total effect of the four comorbidities was found to increase insomnia risk with a relatively large effect (OR = 3.61, 95%CI 2.82 to 4.63).

### Risk factors for insomnia in veterans

#### Sociodemographic factors

**Fig 8:**
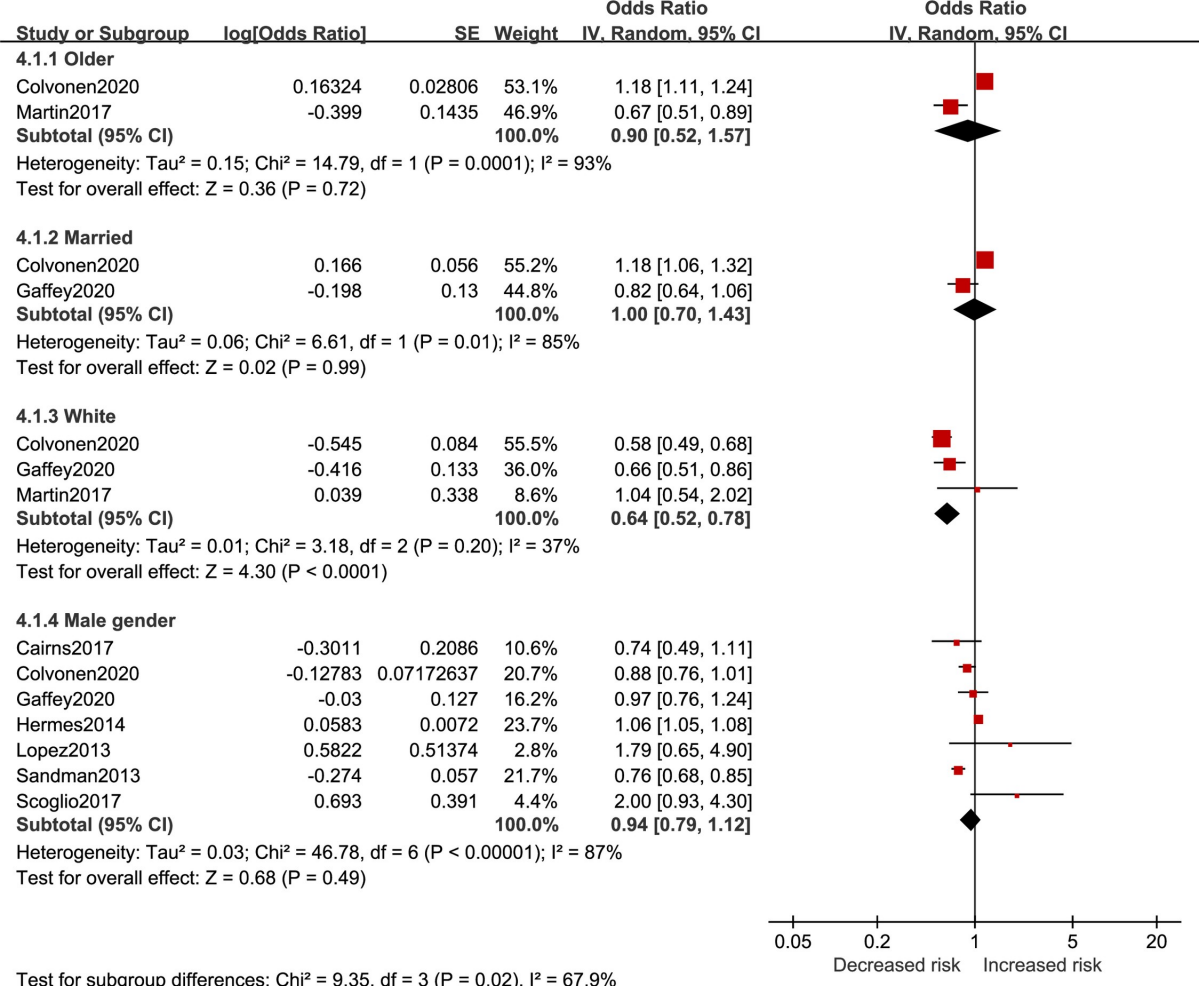
Sociodemographic factors for insomnia in veterans.

Results of sociodemographic factors for insomnia risk in veterans are reported in Fig 8. Being white (OR = 0.64, 95%CI 0.52 to 0.78) was related to lower insomnia risk. Aging (OR = 0.90, 95%CI 0.52 to 1.57), marriage (OR = 1.00, 95%CI 0.70 to 1.43), and male gender (OR = 0.94, 95%CI 0.79 to 1.12) had no significant influence for insomnia risk.

### Army-specific factors

**Fig 9:**
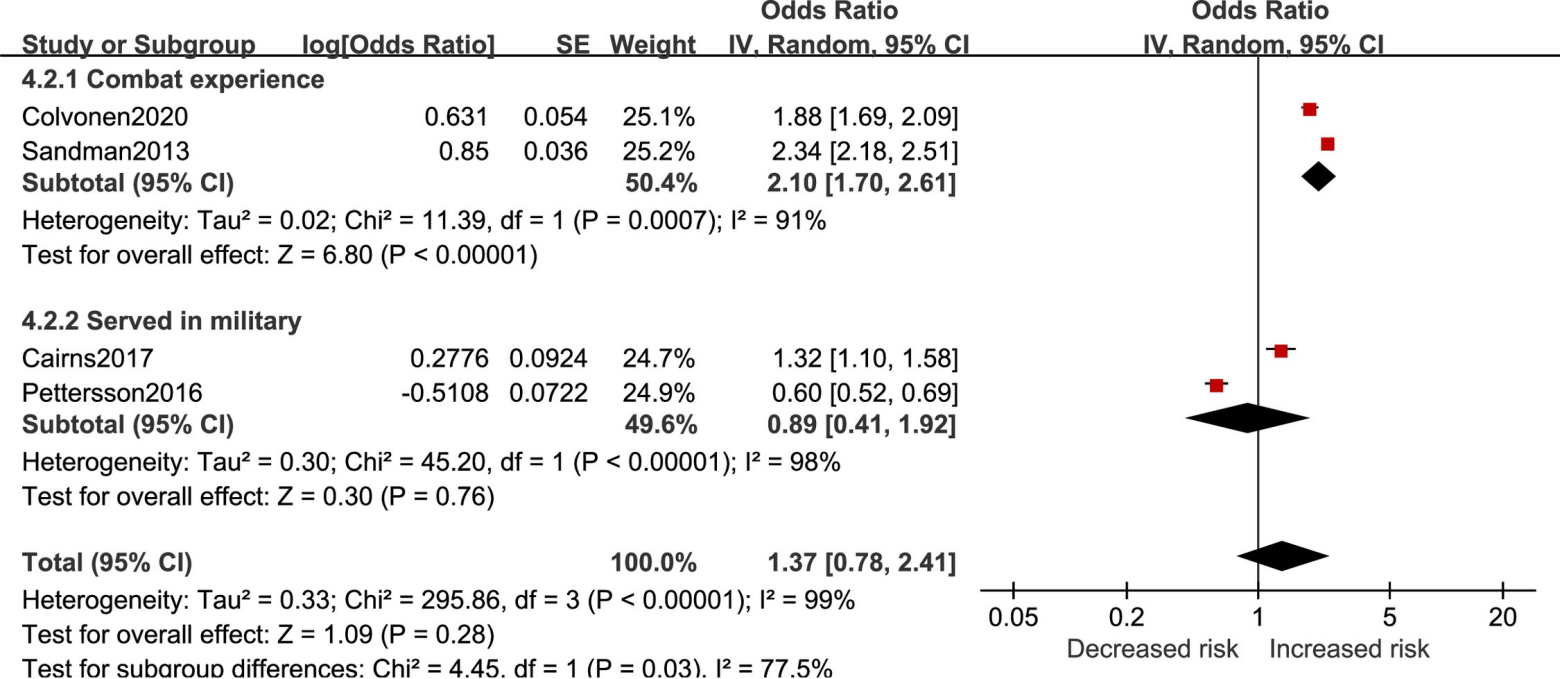
Army-specific factors for insomnia in veterans.

Results of army-specific effect on insomnia risk in veterans are reported in Fig 9. Combat experience was associated with greater risk of developing insomnia (OR = 2.10, 95%CI 1.70 to 2.61). Experience of serving in military had no effect on insomnia risk (OR = 0.89, 95%CI 0.41 to 1.92). The total effect of army-specific showed no significant influence on insomnia risk (OR = 1.37, 95%CI 0.78 to 2.41).

#### Comorbidity factors

**Fig 10:**
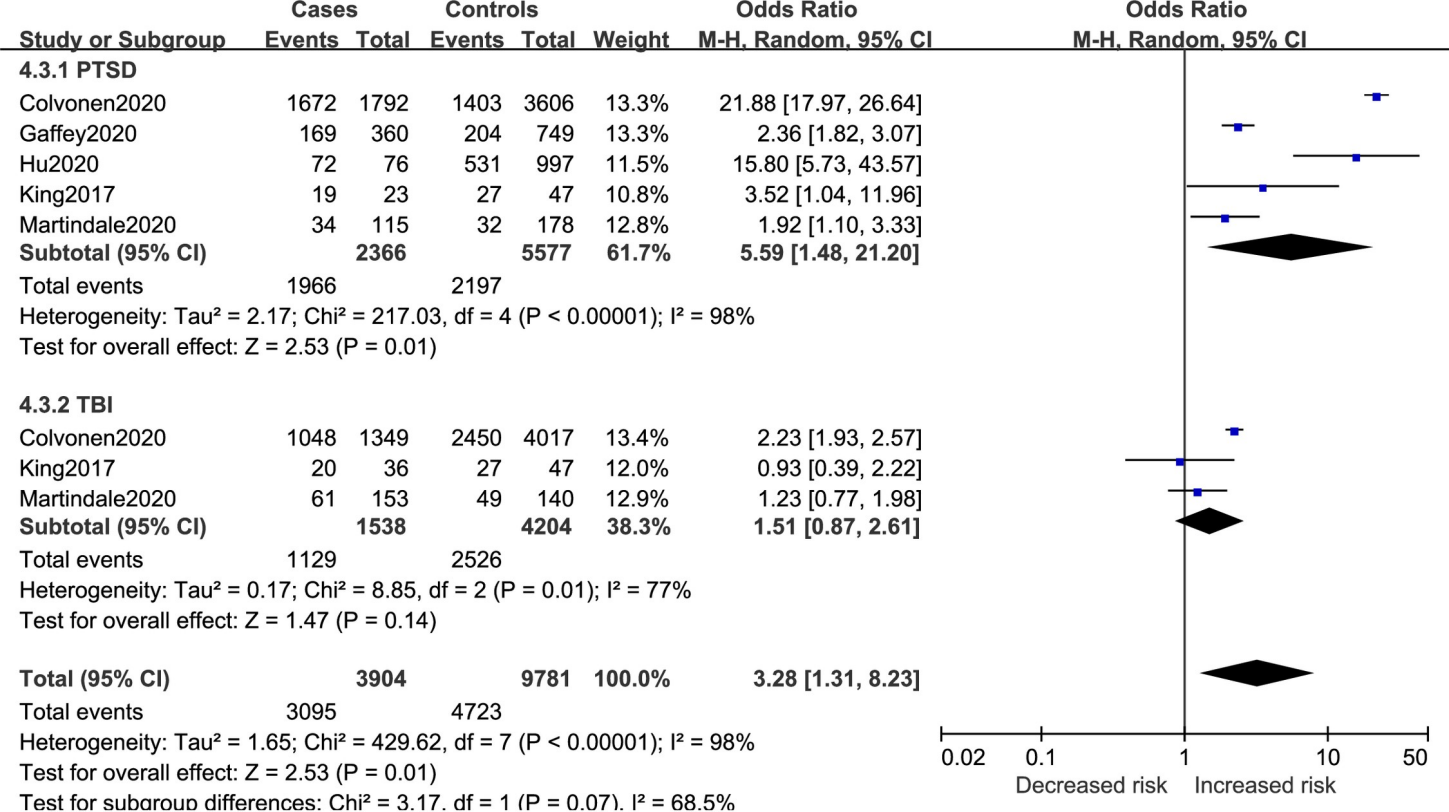
Comorbidity factors for insomnia in veterans.

Results of comorbidities for insomnia risk in veterans are reported in Fig 10. The final merged results revealed that PTSD (OR = 5.59, 95%CI 1.48 to 21.20) could increase insomnia risk while TBI (OR = 1.51, 95%CI 0.87 to 2.61) had no influence on the risk of developing insomnia. The total effect of the two comorbidities demonstrated a tendency to increase insomnia risk (OR = 3.28, 95%CI 1.31 to 8.23).

## Discussion

The pooled outcomes identified four sociodemographic (i.e., overweight/obesity, higher BMI, male gender, >35 years old) and one comorbidity (i.e., depression) risk factors for SDB in active duty personnel; four sociodemographic (i.e., aging, alcohol dependence, white race and female gender), two army-specific (i.e., deployment experience, combat experience), and four comorbidity (i.e., depression, PTSD, TBI and anxiety) risk factors for insomnia in active duty personnel; and one army-specific (i.e., combat experience) and one comorbidity (i.e., PTSD) risk factor for insomnia in veterans.

Gaining more weight (i.e., obesity or higher BMI) was associated with higher SDB risk in active-duty personnel with a medium effect size. This outcome is similar to evidence provided in previous studies and suggests that it is quite important for military personnel to maintain proper weight because it impacts not only their ability to fight but also SDB risk. Alcohol dependence showed higher insomnia risk (OR = 1.76 95%CI 1.4 to 2.21) and a small but significant decrease of SDB risk (OR = 0.94 95%CI 0.92 to 0.96) in active-duty personnel. However, we think more articles are needed in the future to reach a more accurate and convincing conclusion considering that only two articles were included in this latter analysis.

Age plays an important role in the development of soldiers’ retirement policy with the consideration that soldiers could be at higher risk for many disorders including SDB when older than a certain age. In the current analysis, pooled evidence showed 3-times-higher SDB risk for active-duty personnel and 3.64-times-higher SDB risk for veterans older than 35 years old. As soldiers aged, insomnia risk increased in the active-duty personnel group. More studies are needed to confirm the age point where the steepest turning point of risk for SDB or insomnia appears and could help guide policy development.

Merely serving in the military does not increase insomnia risk for veterans. However, deployment experience and combat experience could increase insomnia risk. These outcomes indicate that influences that lead to higher risk of insomnia come from deployment or combat experience rather than military serving experience. Thus, future research should break down deployment and combat experiences into more specific categories of possible influences to figure out which factor plays a more important role in the developing of insomnia.

The effects of comorbidities on insomnia risk in active-duty personnel were quite remarkable. Patients with any of the four comorbidities (i.e., depression, PTSD, TBI, anxiety) were 3.61 times more likely to develop insomnia compared with healthy controls in active-duty personnel. This result suggests more concern or even early preventive treatment should be given to soldiers with these comorbidities even when they do not exhibit relevant symptoms. In contrast, effects of comorbidity factors on SDB risk were mostly insignificant. The only exception is that depression could increase SDB risk in active-duty personnel. However, considering only two to three studies were included in the analysis of comorbidity factors for SDB risk, results of this section should be updated when more studies are available in the future.

Combat experience could bring higher insomnia risk for both of the two groups. However, the effect of some factors on insomnia risk could differ in veteran and active-duty personnel groups. Aging is a risk factor for insomnia in active-duty personnel while no significant result was observed in veterans. White people were less likely to develop insomnia in veterans while the opposite result was observed in active-duty personnel. TBI brought higher insomnia risk in active-duty personnel while no significant effect of TBI was observed in veterans. Three possible factors may explain this situation: First, symptoms of comorbidities (e.g., TBI) caused by military service gradually improve over time after proper treatment. Second, the life and work environments of veterans and active-duty personnel are totally different, so potential influencing factors could serve as mediators to influence the relationship between risk factors and outcomes. Third, the number of included studies is insufficient, and the results may be biased. More in-depth comparison between veteran and active-duty personnel is not yet possible, as many factors could only be analyzed in one group since suitable articles are limited. Data on risk factors for SDB in veterans is most insufficient with PTSD being the only analyzable factor, making the comparison of SDB risk between active-duty personnel and veterans impossible.

### Strengths and limitations

Systematic search strategy was used to get comprehensive records in four databases, and articles of different countries were included. The search terms were identified after careful discussions and consultations among researchers. There was no similar meta-analyses or reviews exploring the risk factors for sleep disorder in a military sample to our knowledge, so this research provided findings of practical significance and could help to guide future research.

Nevertheless, several limitations could not be ignored. More than half of the studies included were cross-sectional, and as a result, the ability to explain the causal relationships between these factors and sleep disorders was restricted. Considering the number of studies could be quite small after sorted into different subgroups, relatively lenient entry criteria were drawn up to ensure a sufficient number of articles to get meaningful results. Consequently, many outcomes were accompanied by high heterogeneity because assessment tools varied across studies. Outliers were identified and excluded to reduce heterogeneity, while further subgroup analysis could not be performed due to the much-detailed grouping in this study.

### Implications

In this preliminary meta-analysis, several risk and protective factors for insomnia and SDB were identified, providing deeper views into detection of those at higher risk of developing sleep disorders in military group and guidance for future research. However, the lack of usable data was quite prominent and there would be an urgent need for prospective cohort studies of risk factors for sleep impairment in the military sample.

## Supporting information

S1 File Protocol registered in PROSPERO. (PDF)

S2 File PRISMA checklist. (PDF)

S3 File Full search strategy. (PDF)

S4 File Quality assessment of the included studies. (PDF)

S5 File Publication bias assessment. (PDF)
