# Supplementary material for: The risk factors for insomnia and sleep-disordered breathing in military communities: A meta-analysis
Source: PLoS One. 2021 May 6;16(5):e0250779. doi: 10.1371/journal.pone.0250779 (PMC8101965; doi:10.1371/journal.pone.0250779)
Supplement: S3 File — (PDF) [file pone.0250779.s003.pdf]

S3: Full search strategy

Database	Syntaxis
PubMed	(((((("sleep problem"[Title/Abstract]) OR "sleep disorder"[Title/Abstract]) OR sleep[Title/Abstract])) AND ((((((((((veteran*) OR soldier*) OR army) OR navy) OR "marine corp") OR troop*) OR "air force") OR armed) OR peacekeeper*) OR defense)) AND ((((risk) OR predictor*) OR prediction) OR predisposition))
Embase	(sleep:ab,ti OR 'sleep disorder':ab,ti OR 'sleep problem':ab,ti) AND (veteran* OR soldier* OR army OR navy OR troop* OR 'air force' OR armed OR peacekeeper* OR defense OR 'marine corp') AND (risk OR predictor* OR prediction OR predisposition)
PsycINFO	(("sleep disorder" or sleep or "sleep problem") and (veteran* or soldier* or army or navy or "marine corp" or troop* or "air force" or armed or peacekeeper* or defense) and (risk or predictor* or prediction or predisposition)).mp. [mp=title, abstract, heading word, table of contents, key concepts, original title, tests & measures, mesh]
Web of Science	#3 AND #2 AND #1<br>#3 TS=(risk OR predictor* OR prediction OR predisposition)<br>#2 TS=(veteran* OR soldier* OR army OR navy OR troop* OR air force OR armed OR peacekeeper* OR defense OR marine corp)<br>#1 TS=(sleep OR sleep disorder OR sleep problem)
