# Supplementary material for: The risk factors for insomnia and sleep-disordered breathing in military communities: A meta-analysis
Source: PLoS One. 2021 May 6;16(5):e0250779. doi: 10.1371/journal.pone.0250779 (PMC8101965; doi:10.1371/journal.pone.0250779)
Supplement: S4 File — (PDF) [file pone.0250779.s004.pdf]

S4 Quality assessment of included studies

Table 1 Quality assessment of cross-sectional studies with AHRQ methodological checklist.

Included study	1	2	3	4	5	6	7	8	9	10	11	Score
Baird et al. 2013	1	1	1	0	0	1	1	0	0	1	0	6
Cairns et al. 2017	1	0	1	0	0	1	0	1	0	0	0	4
Lee et al. 2013	1	0	1	1	0	1	0	0	0	0	0	4
Mysliwiec et al. 2015	1	0	1	0	0	1	1	0	1	1	0	6
Pilakasiri et al. 2018	1	1	1	0	0	1	1	0	0	0	0	5
Iqbal et al. 2016	1	0	0	0	0	1	1	0	0	0	0	3
Mysliwiec et al. 2013	1	1	1	0	0	1	1	0	0	0	0	5
Martindale et al. 2020	1	1	0	0	0	1	1	0	0	0	0	4
Foster et al. 2017	1	1	1	0	0	1	1	0	0	0	0	5
Kanefsky et al. 2019	1	1	0	0	0	1	1	1	0	0	0	5
Hermes et al. 2014	1	0	1	1	0	1	0	0	0	0	0	4
Klingaman et al. 2017	1	1	1	0	0	1	0	0	0	1	0	5
Lopez et al. 2013	1	0	1	0	0	1	0	0	0	0	0	3
Martin et al. 2017	1	0	1	0	1	1	0	1	1	1	0	7
Taylor et al. 2016	1	1	1	1	0	1	0	0	1	1	0	7
Pettersson et al. 2016	1	0	1	0	0	0	0	1	0	1	0	4
Adrian et al. 2018	1	1	1	1	0	1	0	1	0	1	0	7
Colvonen et al. 2020	1	0	1	0	0	1	0	0	0	0	0	3
Mosti et al. 2019	1	1	1	1	0	1	0	0	1	1	0	7
Scoglio et al. 2017	1	0	1	0	0	0	1	0	1	1	1	6
Sandman et al. 2013	1	0	1	1	0	0	0	0	0	0	0	3
Hu et al. 2020	1	1	1	0	0	1	0	1	0	1	0	6
Gaffey et al. 2020	1	1	1	0	0	0	0	0	1	1	1	6

1. Define the source of information (survey, record review)
2. List inclusion and exclusion criteria for exposed and unexposed subjects (cases and controls) or refer to previous publications
3. Indicate time period used for identifying patients
4. Indicate whether or not subjects were consecutive if not population-based
5. Indicate if evaluators of subjective components of study were masked to other aspects of the status of the participants
6. Describe any assessments undertaken for quality assurance purposes (e.g.,test/retest of primary outcome measurements)
7. Explain any patient exclusions from analysis
8. Describe how confounding was assessed and/or controlled.
9. If applicable, explain how missing data were handled in the analysis
10. Summarize patient response rates and completeness of data collection
11. Clarify what follow-up, if any, was expected and the percentage of patients for which incomplete data or follow-up was obtained

Table 2 Quality assessment of cohort studies with NOS methodological checklist.

Included study	Selection 1)	Selection 2)	Selection 3)	Selection 4)	Comparability 1)	Exposure 1)	Exposure 2)	Exposure 3)	Score
Crump et al. 2019	*	*	*		**	*	*	*	8
Langton et al. 2016	*	*	*		*	*	*	*	7
Caldwell et al. 2019	*	*	*	*		*	*	*	7
King et al. 2017	*	*	*				*	*	5
