# Supplementary material for: The risk factors for insomnia and sleep-disordered breathing in military communities: A meta-analysis
Source: PLoS One. 2021 May 6;16(5):e0250779. doi: 10.1371/journal.pone.0250779 (PMC8101965; doi:10.1371/journal.pone.0250779)
Supplement: S5 File — (PDF) [file pone.0250779.s005.pdf]

### 1.1.1 Publication bias of Male gender for SA risk in active duty personnel

Begg's Test

```
adj. Kendall's Score (P-Q) =        0
        Std. Dev. of Score =     2.94
         Number of Studies =        4
                         z   =     0.00
                  Pr > |z| =    1.000
                         z   =    -0.34 (continuity corrected)
                  Pr > |z| =    1.000 (continuity corrected)
```

Egger's test

| Std_Eff | Coef. | Std. Err. | t | P>\|t\| | [95% Conf. Interval] | |
|---|---|---|---|---|---|---|
| slope | .761204 | .1010589 | 7.53 | 0.017 | .3263828 | 1.196025 |
| bias | 1.396758 | 2.11707 | 0.66 | 0.577 | -7.712257 | 10.50577 |

### 1.1.2 Publication bias of overweight/obesity for SA risk in active duty personnel

Begg's Test

```
adj. Kendall's Score (P-Q) =       -1
        Std. Dev. of Score =     1.91
         Number of Studies =        3
                         z   =    -0.52
                  Pr > |z| =    0.602
                         z   =     0.00 (continuity corrected)
                  Pr > |z| =    1.000 (continuity corrected)
```

Egger's test

| Std_Eff | Coef. | Std. Err. | t | P>\|t\| | [95% Conf. Interval] | |
|---|---|---|---|---|---|---|
| slope | 1.171709 | .3745025 | 3.13 | 0.197 | -3.586796 | 5.930214 |
| bias | -12.67983 | 31.13733 | -0.41 | 0.754 | -408.3172 | 382.9575 |

### 1.1.3 Publication bias of higher BMI for SA risk in active duty personnel

Begg's Test

adj. Kendall's Score (P-Q) = -2
Std. Dev. of Score = 2.94
Number of Studies = 4
z = -0.68
Pr > |z| = 0.497
z = 0.34 (continuity corrected)
Pr > |z| = 0.734 (continuity corrected)

Egger's test

| Std_Eff | Coef. | Std. Err. | t | P>\|t\| | [95% Conf. Interval] | |
|---|---|---|---|---|---|---|
| slope | .63298 | .0505691 | 12.52 | 0.006 | .415399 | .8505611 |
| bias | -.1811568 | 2.740194 | -0.07 | 0.953 | -11.97126 | 11.60895 |

### 1.2.1 Publication bias of TBI for SA risk in active duty personnel

Harbord's modified test for small-study effects:
Regress Z/sqrt(V) on sqrt(V), where Z is the efficient score and V is the score variance

.
Number of studies = 3 Root MSE = .3837

| Z/sqrt(V) | Coef. | Std. Err. | t | P>\|t\| | [95% Conf. Interval] | |
|---|---|---|---|---|---|---|
| sqrt(V) | .3433741 | .0066146 | 51.91 | 0.012 | .259328 | .4274202 |
| bias | -1.601022 | .2852649 | -5.61 | 0.112 | -5.225657 | 2.023612 |

Test of H0: no small-study effects P = 0.112

### 2.2.1 Publication bias of PTSD for SA risk in veterans

Harbord's modified test for small-study effects:
Regress Z/sqrt(V) on sqrt(V), where Z is the efficient score and V is the score variance

.
Number of studies = 3 Root MSE = 2.343

| Z/sqrt(V) | Coef. | Std. Err. | t | P>\|t\| | [95% Conf. Interval] | |
|---|---|---|---|---|---|---|
| sqrt(V) | 2.198033 | 1.993343 | 1.10 | 0.469 | -23.1298 | 27.52586 |
| bias | -6.081886 | 6.688416 | -0.91 | 0.530 | -91.06626 | 78.90249 |

Test of H0: no small-study effects P = 0.530

### 3.1.2 Publication bias of alcohol dependence for SA risk in veterans

Begg's Test

adj. Kendall's Score (P-Q) = 3
Std. Dev. of Score = 1.91
Number of Studies = 3
z = 1.57
Pr > |z| = 0.117
z = 1.04 (continuity corrected)
Pr > |z| = 0.296 (continuity corrected)

Egger's test

| Std_Eff | Coef. | Std. Err. | t | P>\|t\| | [95% Conf. Interval] | |
|---|---|---|---|---|---|---|
| slope | .3166207 | .016916 | 18.72 | 0.034 | .101683 | .5315584 |
| bias | 5.293075 | 1.108919 | 4.77 | 0.131 | -8.797075 | 19.38322 |

### 3.1.4 Publication bias of male gender for SA risk in veterans

Begg's Test

```
 adj. Kendall's Score (P-Q) =        -2
         Std. Dev. of Score =      2.94
          Number of Studies =         4
                         z  =     -0.68
                   Pr > |z| =     0.497
                         z  =      0.34 (continuity corrected)
                   Pr > |z| =     0.734 (continuity corrected)
```

Egger's test

```
------------------------------------------------------------------------------
     Std_Eff |      Coef.   Std. Err.      t    P>|t|     [95% Conf. Interval]
-------------+----------------------------------------------------------------
       slope |  -.1786795   .2343015    -0.76   0.525    -1.186797    .8294383
        bias |  -2.595282   2.703334    -0.96   0.438    -14.22679    9.036227
------------------------------------------------------------------------------
```

### 3.2.1 Publication bias of deployment experience for insomnia risk in active duty personnel

Begg's Test

```
 adj. Kendall's Score (P-Q) =         2
         Std. Dev. of Score =      2.94
          Number of Studies =         4
                         z  =      0.68
                   Pr > |z| =     0.497
                         z  =      0.34 (continuity corrected)
                   Pr > |z| =     0.734 (continuity corrected)
```

Egger's test

```
------------------------------------------------------------------------------
     Std_Eff |      Coef.   Std. Err.      t    P>|t|     [95% Conf. Interval]
-------------+----------------------------------------------------------------
       slope |   .2406486   .0807509     2.98   0.097    -.1067944    .5880916
        bias |   1.732308   1.044848     1.66   0.239     -2.76331    6.227927
------------------------------------------------------------------------------
```

### 3.3.1 Publication bias of depression for insomnia risk in active duty personnel

Begg's Test

```
   adj. Kendall's Score (P-Q) =         1
           Std. Dev. of Score =      1.91
            Number of Studies =         3
                           z  =      0.52
                     Pr > |z| =     0.602
                           z  =      0.00 (continuity corrected)
                     Pr > |z| =     1.000 (continuity corrected)
```

Egger's test

| Std_Eff | Coef. | Std. Err. | t | P>\|t\| | [95% Conf. Interval] | |
|---|---|---|---|---|---|---|
| slope | 1.065427 | .0764996 | 13.93 | 0.046 | .0934075 | 2.037446 |
| bias | 6.38049 | 7.375057 | 0.87 | 0.546 | -87.3285 | 100.0895 |

### 3.3.3 Publication bias of TBI for insomnia risk in active duty personnel

Begg's Test

```
   adj. Kendall's Score (P-Q) =         0
           Std. Dev. of Score =      2.94
            Number of Studies =         4
                           z  =      0.00
                     Pr > |z| =     1.000
                           z  =     -0.34 (continuity corrected)
                     Pr > |z| =     1.000 (continuity corrected)
```

Egger's test

| Std_Eff | Coef. | Std. Err. | t | P>\|t\| | [95% Conf. Interval] | |
|---|---|---|---|---|---|---|
| slope | .5825948 | .0092504 | 62.98 | 0.000 | .5427937 | .6223959 |
| bias | -.212819 | 1.927286 | -0.11 | 0.922 | -8.50526 | 8.079622 |

### 3.3.3 Publication bias of TBI for insomnia risk in active duty personnel

Begg's Test

```
   adj. Kendall's Score (P-Q) =         1
           Std. Dev. of Score =      1.91
            Number of Studies =         3
                           z   =      0.52
                    Pr > |z| =     0.602
                           z   =      0.00 (continuity corrected)
                    Pr > |z| =     1.000 (continuity corrected)
```

Egger's test

| Std_Eff | Coef. | Std. Err. | t | P>\|t\| | [95% Conf. Interval] | |
|---|---|---|---|---|---|---|
| slope | 1.241792 | .0291441 | 42.61 | 0.015 | .8714812 | 1.612104 |
| bias | 3.860566 | 5.611757 | 0.69 | 0.616 | -67.44356 | 75.16469 |

### 4.1.3 Publication bias of white race for insomnia risk in veterans

Begg's Test

```
   adj. Kendall's Score (P-Q) =         3
           Std. Dev. of Score =      1.91
            Number of Studies =         3
                           z   =      1.57
                    Pr > |z| =     0.117
                           z   =      1.04 (continuity corrected)
                    Pr > |z| =     0.296 (continuity corrected)
```

Egger's test

| Std_Eff | Coef. | Std. Err. | t | P>\|t\| | [95% Conf. Interval] | |
|---|---|---|---|---|---|---|
| slope | -.7379556 | .015854 | -46.55 | 0.014 | -.9394002 | -.5365109 |
| bias | 2.338831 | .1316963 | 17.76 | 0.036 | .6654706 | 4.012192 |

### 4.1.3 Publication bias of male gender for insomnia risk in veterans

Begg's Test

```
   adj. Kendall's Score (P-Q) =          5
             Std. Dev. of Score =       6.66
               Number of Studies =          7
                               z   =       0.75
                       Pr > |z| =     0.453
                               z   =       0.60 (continuity corrected)
                       Pr > |z| =     0.548 (continuity corrected)
```

Egger's test

| Std_Eff | Coef. | Std. Err. | t | P>\|t\| | [95% Conf. Interval] | |
|---|---|---|---|---|---|---|
| slope | .0632548 | .0225763 | 2.80 | 0.038 | .0052205 | .1212892 |
| bias | -1.304175 | 1.203361 | -1.08 | 0.328 | -4.397512 | 1.789163 |

### .4.3.1 Publication bias of PTSD for insomnia risk in veterans

Harbord's modified test for small-study effects:
Regress Z/sqrt(V) on sqrt(V), where Z is the efficient score and V is the score variance

.
Number of studies = 5 Root MSE = 4.222

| Z/sqrt(V) | Coef. | Std. Err. | t | P>\|t\| | [95% Conf. Interval] | |
|---|---|---|---|---|---|---|
| sqrt(V) | 2.411393 | .3457062 | 6.98 | 0.006 | 1.311202 | 3.511585 |
| bias | -5.238919 | 3.017782 | -1.74 | 0.181 | -14.84285 | 4.365011 |

Test of H0: no small-study effects P = 0.181

## 4.3.1 Publication bias of TBI for insomnia risk in veterans

Harbord's modified test for small-study effects:
Regress Z/sqrt(V) on sqrt(V), where Z is the efficient score and V is the score variance

.
Number of studies = 3 Root MSE = .4795

| Z/sqrt(V) | Coef. | Std. Err. | t | P>\|t\| | [95% Conf. Interval] | |
|---|---|---|---|---|---|---|
| sqrt(V) | .8972334 | .0487195 | 18.42 | 0.035 | .2781939 | 1.516273 |
| bias | -2.500315 | .4460618 | -5.61 | 0.112 | -8.168067 | 3.167438 |

Test of H0: no small-study effects P = 0.112
